# State-of-the-Art Methods for Skeletal Muscle Glycogen Analysis in Athletes—The Need for Novel Non-Invasive Techniques

**DOI:** 10.3390/bios7010011

**Published:** 2017-02-23

**Authors:** Jacob Greene, Julien Louis, Olga Korostynska, Alex Mason

**Affiliations:** 1 Department of Built Environment, Faculty of Engineering and Technology, BEST Research Institute, Liverpool John Moores University, Liverpool L3 3AF, UK; J.Greene@2013.ljmu.ac.uk; 2 Faculty of Science, School of Sports and Exercise Science, Liverpool John Moores University, Liverpool L3 3AF, UK; J.B.Louis@ljmu.ac.uk; 3 Department of Civil Engineering, Faculty of Engineering and Technology, Liverpool John Moores University, Liverpool L3 3AF, UK; O.Korostynska@ljmu.ac.uk; 4 Animalia, Norwegian Meat and Poultry Research Centre, Økern 0513, Oslo, Norway; 5 Department of Built Environment, Faculty of Engineering and Technology, Liverpool John Moores University, Liverpool L3 3AF, UK

**Keywords:** muscle glycogen, carbohydrates, muscle biopsy, magnetic resonance spectroscopy, musculoskeletal ultrasound, electromagnetic sensors

## Abstract

Muscle glycogen levels have a profound impact on an athlete’s sporting performance, thus measurement is vital. Carbohydrate manipulation is a fundamental component in an athlete’s lifestyle and is a critical part of elite performance, since it can provide necessary training adaptations. This paper provides a critical review of the current invasive and non-invasive methods for measuring skeletal muscle glycogen levels. These include the gold standard muscle biopsy, histochemical analysis, magnetic resonance spectroscopy, and musculoskeletal high frequency ultrasound, as well as pursuing future application of electromagnetic sensors in the pursuit of portable non-invasive quantification of muscle glycogen. This paper will be of interest to researchers who wish to understand the current and most appropriate techniques in measuring skeletal muscle glycogen. This will have applications both in the lab and in the field by improving the accuracy of research protocols and following the physiological adaptations to exercise.

## 1. Introduction

Over recent decades, technology has evolved in the professional sporting environment, with ever more acceptance that meticulous attention to detail can make the difference between winning and losing. However, the process of collecting accurate data requires adapted equipment and methods are often expensive and invasive, such as muscle biopsies to estimate glycogen concentration or venepuncture to estimate blood minerals and lactate. Athletic performance can translate into many other areas where optimal human performance is not only a fundamental requirement but also a necessity. Aerospace, military, aircraft, medical, and many more personnel all need to be at peak physical and mental performance to ensure optimal results. Sensor technology which can constantly monitor the biology of an individual in such extremely taxing situations will ensure that errors are limited, making a vital difference in the ever-advancing world. 

Advances in technology have permitted endurance athletes, sports teams, and physicians to monitor player movements [1], workloads [2], and biometric indicators [3] enabling optimisation of athlete performance and also reducing the risk of injury. To date, there are a few technologies that allow the analysis of real-time physiological factors. The sensors in this research allow further data to be collected for individual physiological player monitoring. Sensors that determine physiological response to changes in competition and training allow many benefits for sports practitioners such as nutritionists, injury rehab professionals, and strength and conditioning coaches, etc. This ability undoubtedly will lead to a more individualized approach for the athletes, which is vital at the elite end of the performance spectrum. 

There has been some work demonstrated in literature regarding the measurement of skeletal muscle glycogen. However, the existence of convenient non-invasive real-time measurement techniques remains elusive. In addition, there has never been a comprehensive review of the available technologies for such measurements. Therefore, this paper aims to critically review the current state of the art in this area, and identify promising techniques that could achieve non-invasive and real-time monitoring.

## 2. Importance of Muscle Glycogen

**Figure 1:**
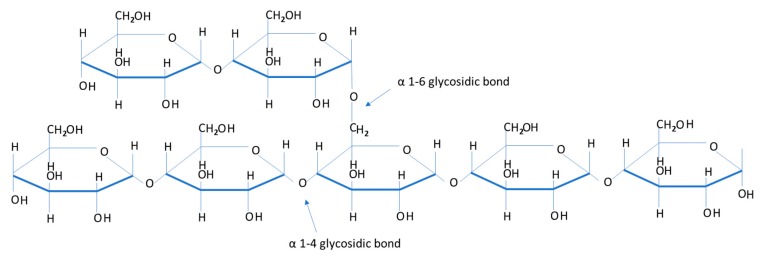
A section of a glycogen molecule illustrating individual glucosyl units. It shows the two different types of glycosidic bonds used to make up glycogen.

Muscle glycogen provides the main source of energy during anaerobic exercise. Furthermore, total glycogen stores within the body also contribute significantly to energy metabolism in endurance-type events lasting longer in duration. Therefore, endurance based events require strategic preparation of carbohydrate (CHO) based fuels (muscle and liver glycogen, blood glucose and blood muscle, and liver lactate) to sustain the high demands for energy production [4,5,6]. Glycogen and the enzymes responsible for glycogen synthesis (glycogenesis) are contained within the cytoplasm of liver and muscle cells. Excess glucose under normal circumstances following the ingestion of carbohydrate, enters the pathways of energy metabolism where it is either stored as glycogen or converted to fat. Glycogenesis is the formation of glycogen from glucose. The demand for glucose and ATP (adenosine triphosphate) depends on the rate that glycogen is synthesized. If both glucose and ATP are present in substantial amounts, then the surplus of insulin stimulates glycogenesis for storage in the liver and muscle cells. Glycogen is the principal d-glucose storage polymer in humans. Most human cells have glycogen, but only liver and skeletal muscle cells are able to store significant quantities of this molecule [7]. Glycogen is a polysaccharide, composed of hundreds of glucose molecules (monosaccharides) joined end to end, with prevalent branches. Osmotic pressure is dependent on the number, not the size, of dissolved substances (Figure 1). 

A single glycogen molecule may contain 5000 glucose units compared to that of 5000 individual glucose molecules. This explains why glycogen is a convenient way to store glucose inside cells without affecting cell osmotic pressure [8]. Glycogen contains a number of OH groups, which allows for the interaction with water in the cell, this means that in terms of weight, glycogen is a substantial fuel [9]. Rates of post-exercise glycogen synthesis are integral to an athlete’s regime, this allows the athlete to ensure sufficient energy stores for the following day, a fundamental component in events which take course over many days. Without consumption of CHO post-exercise, glycogen synthesis occurs at rates of 1–2 mmol/kg wet weight (w.w.) of muscle/h through gluconeogenesis [10]. However, when large amounts of CHO are consumed post-exercise, glycogen synthesis improves greatly, rates of resynthesis increase to within the range of 5–10 mmol/kg w.w./h and then continue throughout the recovery stage [4]. Maximising muscle glycogen synthesis in-between important exercise sessions and events therefore is dependent on matching fuel stores closely with the demands of the intended exercise intensity and duration. 

**Figure 2:**
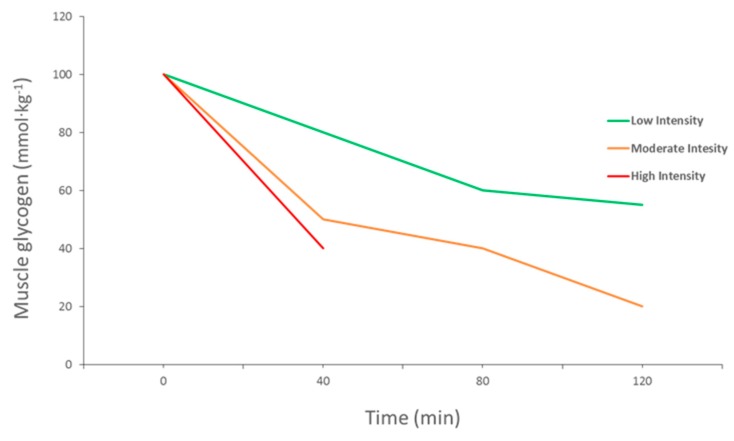
Muscle glycogen use during exercise at different intensities (adapted from Gollnick et al., 1974).

**Figure 3:**
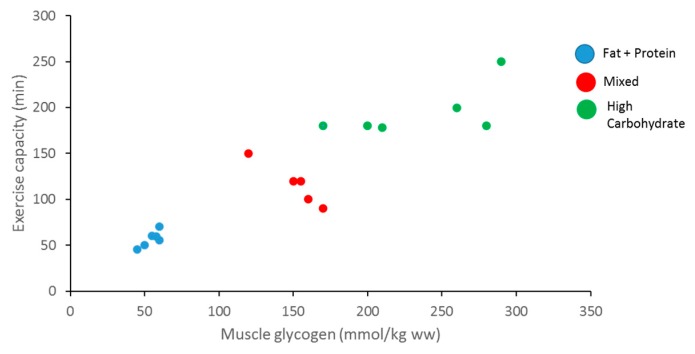
Relationship between muscle glycogen content, exercise capacity, and diet (adapted from Bergstrom et al., 1967).

Carbohydrates are an important source of fuel and energy for intense and prolonged bouts of exercise. Glycogen is one of the main energy sources for ATP production to facilitate muscle contraction during a wide range of exercises, from brief high-intensity exercises to endurance exercises [11]. It has been reported that muscle glycogen content is associated with muscle performance and its depletion by high-intensity exercise leads to a decline in performance, also known as muscle fatigue [12,13] (Figure 2). Glycogen stores in human muscle and liver are determined and will vary dependent upon the individual’s activity status and how much CHO they consume [4]. Normal levels of muscle glycogen stores for a well-trained athlete can usually fuel sporting activity for up to 60–90 min [14]. Muscle glycogen levels have a profound impact on an athlete’s sporting performance, thus measurement is vital (Figure 3). Although fatigue is a complex process involving many variables, there is a large amount of evidence to suggest that the main cause of fatigue during endurance exercise is reduced muscle glycogen and blood glucose availability, which reduces the availability of substrate required to maintain the high CHO oxidation rates necessary to sustain high power outputs [15]. During exercise, CHO availability to the working muscle and central nervous system could become compromised due to the athlete exceeding endogenous stores of CHO when fuel cost is more than expected during either training or competition, reducing performance [16].

The promotion of high CHO availability for prolonged exercise is widely established [17] to ensure there is enough muscle substrate to match the demands of the intensities and volume of endurance training and competition. To do this, one of the most regular requests by a nutritionist or coach to an athlete is to undergo CHO loading to super-compensate muscle and liver glycogen stores in the days before a major endurance competition. As well as ensuring a diet high in CHO, during competition the athlete will also be advised to ingest drinks, bars, and gels with a high CHO content [17]. Muscle glycogen is widely recognised as the primary fuel source for sustaining contractile activity in human skeletal muscle [11] Thus, the ability of skeletal muscle to perform repeated contractions (exercise) is seriously compromised when muscle glycogen reserves reach low levels [11,18,19] demonstrating a clear association between muscle glycogen and fatigue resistance during both prolonged and high-intensity exercise. To this end, it is widely recommended that exercise should be undertaken with high carbohydrate (CHO) availability in order to optimise performance and delay fatigue [20]. Additionally, recent research has also demonstrated that deliberately reducing the CHO availability around training sessions (i.e., by using fasted training, sleeping low, recovering low, training twice a day) is also shown to upregulate the physiological adaptation to training [20]. Consecutively, the concept of CHO periodisation has been introduced to help athletes both enhance the adaptive response to training (through low CHO availability around certain training sessions) and enhance exercise capacity in competition (through high CHO availability during all competitions) [21,22].

## 3. Current Athlete Recommendations

Given the high training loads of elite athletes, traditional nutritional guidelines have typically advised a high carbohydrate (CHO) diet in addition to exogenous CHO provision during exercise and within the immediate recovery period following exercise [20]. However, research gathered over the last decade has established that systematically commencing exercise with low muscle glycogen and limiting CHO intake during exercise supplements a number of markers of mitochondrial biogenesis [17]. Current recommendations involve the periodization of carbohydrates, alternating periods of low or high CHO availability according to the training load [23]. As such, most recent guidelines for CHO intake for training and competition [20] recognise that there is a need for a flexible and individual approach to the intake of CHO, dependent on such factors as training status, type of training and the time to competition. Current guidelines are seen to promote a sliding scale of CHO intake with the goal of matching the predicted energy expenditure of the athletes training and recovery [24]. This research therefore shows the need to be able to monitor an athlete’s glycogen stores to suit the specific needs of the athlete during the cycles of training and competition. 

Untrained individuals consuming a mixed diet normally have a skeletal muscle glycogen content of ~80–90 mmol·kg^−1^, however for athletes involved in regular endurance training; this amount is higher at around 125 mmol·kg^−1^ [25]. As 1 g of glycogen is usually stored with 2–3 g of water, a negative of glycogen loading is that the athletes body mass will likely increase by around 102% after a period of several days CHO ‘loading’ [17]. CHO loading is the process endurance athletes undertake prior to competition to super-compensate glycogen stores to reduce the effects of muscle glycogen depletion on fatigue and exercise capacity [11]. A practical example of CHO loading is reported by Bussau et al. [26] who reported that elevated muscle glycogen stores may be achieved in as little as 24–36 h of rest and high CHO intake (8–12 g·kg·day^−1^), which is a strategy for athletes which are participating in weekly cycles of competition. Being able to monitor glycogen stores during real time will allow development of strategic training programs which cater for specific needs of athletes. Recent studies which used a variety of strategies to reduce CHO stores manipulating CHO availability both endogenously and/ or exogenously during short term training interventions have reported strong upregulation of training adaptation including increased whole body fat oxidation and increased activities of oxidative enzymes, when they were compared with exercising with normalized glycogen stores and high CHO availability [14,27,28] as well as increasing whole-body and intramuscular lipid oxidation [14,29]. 

The practical application of training with lowered CHO availability (typically called “train low”) is still in its early states as there are known limitations and risk factors associated with training consistently with low CHO stores. Training repeatedly with low CHO stores is reported to lead to an inability to maintain the preferred training intensity [14,29] this could furthermore lead to a substandard training impulse (i.e., volume × intensity). CHO restriction during training which is of high-intensity or long in duration can also have negative effects on athlete’s health, making them more susceptible to illness and infection, this is due to the role CHO has in offsetting exercise-induced immunosuppression [30]. Another factor to consider is the increase of muscle protein breakdown, especially with conditions of low muscle glycogen [31]. The advantages and limitations of altering CHO stores throughout training has widely become one of the most debated topics for athletes, coaches, nutritionists, and scientists. The importance of being able to record and measure CHO stores, therefore, is essential to provide real time non-invasive data, providing practical methods for real world situations. 

## 4. Methods of Measuring Muscle Glycogen in Athletes

### 4.1. The Elusive Gold Standard 

**Figure 4:**
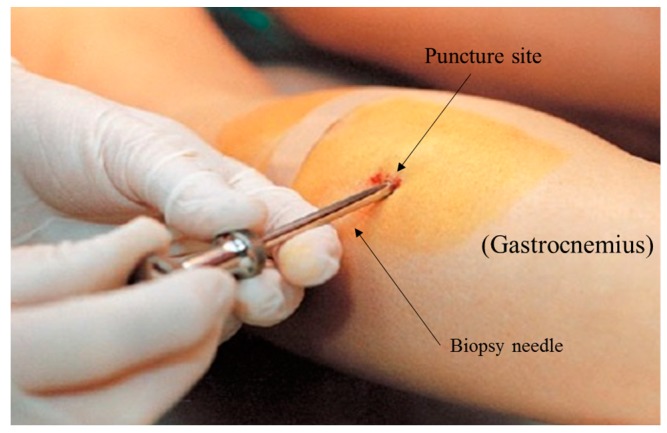
Illustration of an invasive muscle biopsy being performed on the gastrocnemius muscle.

Currently the typical method to measure muscle glycogen requires an invasive muscle biopsy. Involving the use of needles, muscle biopsies have been the standard method to measure muscle glycogen. This procedure is common in sports science but does have its drawbacks due to its invasive nature. The percutaneous biopsy technique is known to obtain skeletal muscle tissue specimens from human subjects. Duchenne (1806–1875) is recognised for the construction of the first needle with a trocar to obtain skeletal muscle from living subjects using this biopsy method [32]. Bergström in the 1960s developed a needle similar to that previously used by Duchenne [33,34]. The modified Bergström technique which is still being used today was developed in the 1980s by Evans et al. [35]. This technique uses the addition of suction (700 TORR) to the inner bore of the biopsy needle after the needle has been inserted into the subject’s muscle (Figure 4). The suction is designed to pull the surrounding muscle tissue into the needle, consequently insuring the taking of a larger piece (X = 78.5 mg) [35]. The advantages of this technique eliminate the need for recurring biopsies because of inadequate muscle sample size and improve the validity of subsequent analysis procedures [36], thus making it a recognised method in clinical and biomedical research environments. 

The modified Bergström is invasive, but it ensures that it is causes as little damage as possible, making the procedure relatively safe. The technique elevates the quality of the sample collected during the testing, whilst doing so under minimal time restraints for what is needed. Multiple biopsies can be taken from one subject during that specific session and the procedure can be completed quickly when the correct preparation is in place [32], this allows for pre-, mid-, and post-exercise biopsies to be taken. Another advantage of biopsies is it allows the measurement of many different outcome variables, not only the analysis of muscle glycogen stores, being useful when other parameters are investigated. Other outcome measures include for example, fibre typing, muscle damage, different fuel substrate stores, mitochondrial biogenesis and respiration, enzyme activity, shifts in metabolites, and protein synthesis [32].

**Figure 5:**
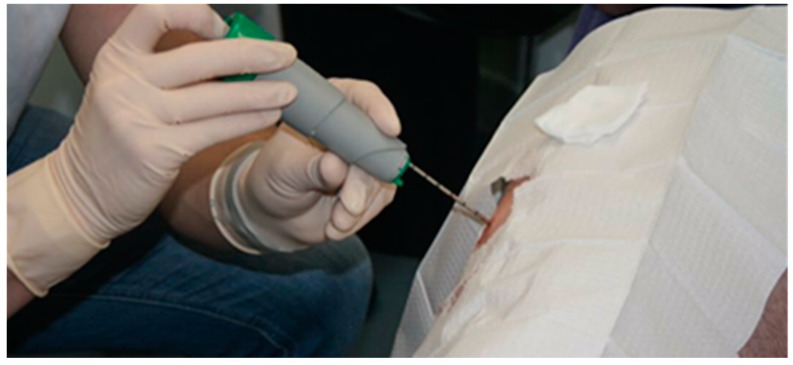
Illustrates the Monopty 12 G, disposable core biopsy instrument (BARD, Brighton, UK) being used on an athlete’s vastus lateralis.

A current example of a muscle biopsy needle that is used in a sporting context (Figure 5) is Monopty 12 G, disposable core biopsy instrument (BARD, Brighton, UK), this needle has been used to provide field data in professional rugby players to measure muscle glycogen utilisation pre- and post-game [37]. Once the biopsy has been taken, it is then essential to immediately snap freeze in liquid nitrogen and store at -80 °C for later analysis. The delay in time and resources that is required to gain a true muscle glycogen reading demonstrates why a non-invasive sensor could provide a practical and time saving method to the professional world of elite performance. 

Although the use of biopsies is relatively safe, practicality in a sports setting, regular measurement of skeletal muscle for the analysis of CHO stores is limited due to its invasive nature. Indeed, biopsies largely take place within a biomedical research setting to limit the risk of infection. After the athlete has undergone a biopsy, it usually takes up to 5–7 days for soreness and swelling to fully dissipate. Although it is very rare, infection can accrue post procedure due to several factors. Tarnoplsky et al.’s research took 13,914 biopsies in both adults and children, with a total of 22 complications throughout [38]. Complications were as follows, local skin infections (eight cases), arterial bleed (two cases), ecchymosis/hematoma (two cases), pain persisting for more than three days (five cases), and a small area local numbness distal to the biopsy (five cases) [38]. 

Most subjects experience local soreness and stiffness in the leg for two or three days after the biopsy, similar to a deep bruise. Therefore, performing biopsies before competition can be difficult to attain due to the disruptions it can cause for the athlete. On occasions, a small lump of scar tissue may form under the site of the incision, but this normally disappears within two to three months, or within a few weeks if correctly massaged, often leaving a small visible scar. There is the possibility of numbness around a small area of the biopsy site, this usually resolves over five to six months. Furthermore, there is a very low risk (estimated at less than 1/5000) of damage to small nerve branches within the muscle, resulting in partial weakness of the muscle and would likely have no impact on day-to-day activities. Nerve injuries in this case usually resolve in 8–12 months, but there is a small risk of minor leg weakness.

### 4.2. Histochemical Methods

Once muscle biopsy samples have been removed, standard procedure requires the sample be immediately frozen in liquid nitrogen and stored at -80 °C. Collagen, blood, and other non-muscle fibre materials are then removed from the sample from under a microscope by a trained lab technician. The samples of muscle fibre (2–3 mg) are then weighed and 500 µL of 1 mol hydrochloric acid/L are added. After heating for 3 h at 100 °C to hydrolyse the glycogen to glycosyl units and cooling down to room temperature, the solution is then neutralized by adding 267 mL tris/KOH. To conclude the procedure, 150 µL is then analysed for glucose using a calibrated specialised glycogen assay kit [39]. After a muscle biopsy has been performed and the sample is prepared to use, glycogen content can be measured by biochemical techniques; however, such techniques are most often performed on muscle homogenates, and can therefore not discriminate between intramyocellular and extramyocellular glucose stores, nor do they allow for muscle fibre typing. In order to measure only intramyocellular energy stores and to differentiate between the different fibre types, histochemical methods have been extensively used [40]. 

**Figure 6:**
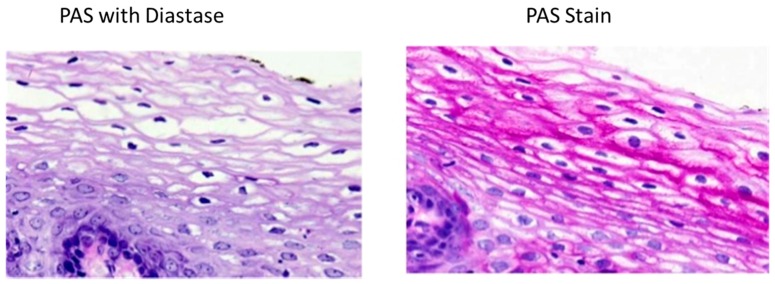
The presence of glycogen is shown by the loss of staining after enzyme treatment when compared to the untreated segments.

Periodic Acid-Schiff (PAS) stain (Figure 6) is based on the reaction of periodic acid with the diol functional groups in glucose and other sugars, oxidizing them to form aldehyde, which in turn reacts with the Schiff reagent to give a purple/magenta stain [40]. PAS stain is therefore not specific to glycogen; it also stains glycoproteins and proteoglycans. In order to single out glycogen form the other PAS-reactive cellular components cryosections can be pre-treated with the glycogenolytic enzyme diastase [7]. The downside is that many published studies do not use this process and therefore glycogen content can be overestimated [40]. A study by Fairchild & Fournier [41] revealed that thawing and air drying muscle cryosections results in glycogen degradation. However, it is common practice in laboratories, where histochemical measurements of glycogen in tissue cryosections are done by PAS staining, to still thaw and dry tissue cryosections after cutting and before fixation [40]. New research which enables laboratories to optimize skeletal muscle preservation and increased stain specificity is to use the monoclonal anti-glycogen IgM antibody [42]. For optimal preservation of glycogen stores, muscle cryosections should not be air-dried. Any cycle of freezing/thawing should be avoided due to the resulting effect in loss of glycogen particles [41]. To increase the specificity of the glycogen staining, use of a monoclonal antibody is recommended by Prats et al. [40]. 

### 4.3. Magnetic Resonance Spectroscopy 

**Figure 7:**
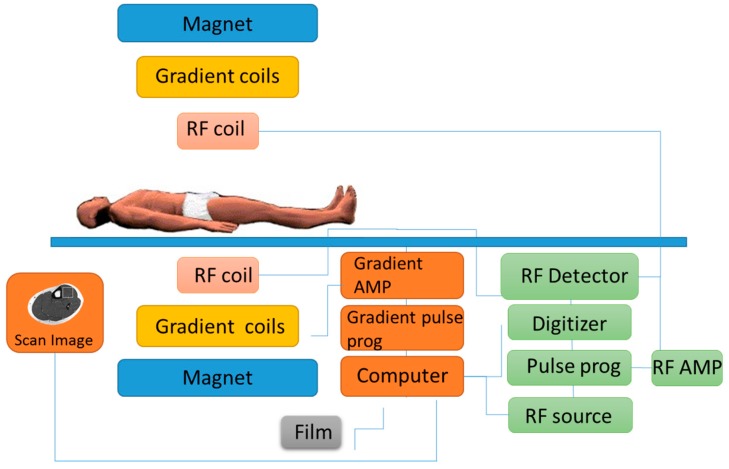
Example of an MRI machine illustrates how the subject is required to be fully stationary during the procedure.

Magnetic resonance spectroscopy (MRS) is usually done alongside the more commonly used magnetic resonance imaging (MRI) scan. MRS measures the chemical content of MR-visible nuclei, which include the metabolically elements of hydrogen (^1^H), carbon (^13^C), and phosphorus (^31^P) [43]. Whereas MRI establishes the spatial distribution of water (and lipid) protons within the site of interest [43]. MRS is performed using the same machine as conventional MRI scanner, using a powerful magnet, radio waves, and a computer to create detailed images (Figure 7). Spectroscopy is a series of tests that are added to the MRI scan across specific regions of the body for chemical metabolism. There are no known health risks associated with the magnetic field or the radio waves used in either MRI or MRS and all contrast agents used are all deemed safe and are FDA-approved. 

During the procedure, a radiology technologist will perform the test in the MRI suite in a hospital’s radiology department or an outpatient imaging center. MRS is used to non-invasively measure tissue glycogen by either using ^13^C natural abundance levels, or ^13^C atoms incorporated into glycogen by ^13^C substrate received through ingestion or intravenous administration. The other method is to use the water signal with chemical exchange saturation transfer imaging (glycoCEST) [44,45]. Recent advances over the last two decades within the field of MRS technology now allow detection of changes in a variety of different intramuscular fuel sources, such as muscle glycogen, non-invasively [46,47,48]. When access to MRS is available, it can be a useful tool to measure athlete’s physical condition during valuable times in their calendar (pre-season, mid-season, end of season), this allows for the evaluation of the athlete’s performance. 

The need for extremely accurate methods to detect small changes in glycogen levels began when it was discovered that in diabetic subjects, responses to physiologic hyperinsulinemia caused changes in glycogen concentrations which were too small to be detected by the current biopsy techniques [49]. It was recognised that this was firstly done by obtaining the ^13^C nuclear magnetic resonance (NMR) spectra of human muscle glycogen in vivo from the 1.1 percent carbon nuclei that naturally occurs as this isotope [50]. Furthermore, NMR measurements of glycogen concentrations can be made more accurate by infusing ^13^C-enriched glucose [51]. ^13^C nuclear magnetic resonance (NMR) spectroscopy was validated by Taylor et al. [48], the study compared the NMR to muscle biopsies and direct biochemical assays for glycogen concentrations. The results reported that in vivo, ^13^C-NMR measurement of human muscle glycogen can be considered just as accurate as biopsy results as well as deliver a higher precision measurement than a biopsy with a direct biochemical assessment. This technology is now proven to allow for the non-invasive method to analyse muscle glycogen, it has fast time resolution making for fast results, can be repeated as many times necessary, and provides very accurate timing. However, gaining access to this expensive specialised equipment is limited and MRS does not have the ability to distinguish between muscle fibre types, MRI machines are also not portable which makes use in an athletic situation difficult. 

### 4.4. Musculoskeletal High Frequency Ultrasound 

**Figure 8:**
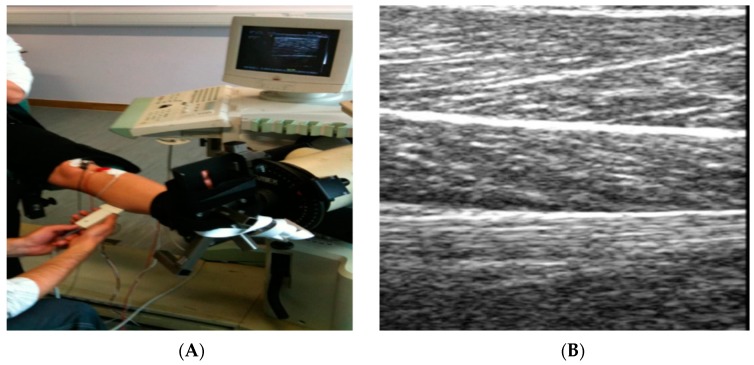
(**A**) Application of ultrasound and equipment involved; (**B**) Example of a grey scale image produced by an Ultrasound when placed directly upon skeletal muscle.

Ultrasound has functioned as a valuable imaging modality in medicine for many decades, in more recent years it has gained increasing practical application and attention in the area of sports medicine. Ultrasound is currently established for evaluating cardiovascular status among athletes, musculoskeletal pathology diagnosis, therapeutic interventions, and to monitor real-time movement of muscles and tendons (Figure 8a) [52,53]. Musculoskeletal ultrasound has established more promise as a point of care device to use within the field, rather than having to incorporate specialised laboratories and technicians. Musculoskeletal ultrasound is not only being utilised as a diagnostic tool but it also has a therapeutic use in treating a vast range of different musculoskeletal conditions affecting athletes [53]. Furthermore, ultrasound velocity now allows for the possible detection of hydration status [54]. This technique was used in a recent study accessing changes in hydration status among National Collegiate Athletic Association Wrestlers, the protocol had the wrestlers to undergo acute bouts of dehydration followed by a 2-h rehydration period. The results demonstrated the potential use of ultrasound technology being deployed as a means of assessing field-based hydration status of athletes [55]. In comparison to the aforementioned methods, ultrasound technology prompts a practical solution in providing a non-invasive and relatively cheap alternative procedure to detect muscle glycogen. This technique determines muscle glycogen content within the muscles by detecting the variations in a grey scale image (Figure 8b), accessing the association between water content and glycogen values [56]. Ultrasound technology is used frequently in medicine and has promising advantages compared to the previous techniques mentioned within this review, such as portability, low cost, no harmful ionizing radiation, real time, and also causes no discomfort or any long-term side effects. 

Recently, methods have been designed to try and overcome the invasive nature of biopsies. MuscleSound^©^ have attempted to design software to enable the ability to use ultrasound to measure skeletal muscle glycogen. MuscleSound^©^ methodology is based upon the measurement of the water content which is associated with glycogen in the muscle. When muscle glycogen is high, the ultrasound image is hypoechoic (dark), and when used for a muscle which has low stores of glycogen and has water loss, the image is hyperechoic (brighter) [57]. The idea behind the MuscleSound^©^ software is to then quantify the observed changes in muscle glycogen levels using image processing and analysis through segmentation of the area that is of interest and measurement of the mean signal intensities [57]. 

Research by Nieman et al. [57] assessed the use of the ultrasound method using a high resolution GE LOGIQ-e ultrasound machine (GE Healthcare, Milwaukee, WI, USA) alongside MuscleSound^©^ software for the ability to measure exercise-induced changes in skeletal muscle glycogen content. Well-trained cyclists endured in a 75-km cycling time trial. Muscle biopsy samples and ultrasound measurements were acquired pre- and post-exercise. Ultrasound images were pre-processed to isolate the muscle area under analysis, with the mean pixel intensity averaged from the three scans and scaled (0 to 100 scale) to create the glycogen score. Pre- and post-exercise muscle biopsy samples were acquired at the vastus lateralis location using the suction-modified percutaneous needle biopsy procedure, and analyzed for glycogen content. MuscleSound^®^ change scores attained from an average of three ultrasound scans at the vastus lateralis site correlated significantly with change in vastus lateralis muscle glycogen content [57]. The data found in this specific study showed that MuscleSound^®^ methodology was able to accurately and non-invasively estimate exercise-induced decreases in vastus lateralis skeletal muscle glycogen content.

However, further research is still needed to ensure this is a viable method to measure muscle glycogen in athletes under several variations. Recent examination of this technique by Bone et al. [56] reported that ultrasound technology failed to measure indirect estimates of muscle glycogen concentrations. The study aimed to validate ultrasound technology for the measurement of muscle glycogen concentrations in well-trained individuals under different conditions which were previously tested by Neiman et al. These conditions included normal glycogen levels, depleted glycogen levels, and loaded levels of glycogen. In addition, creatine loading was consumed by some subjects to provide a possible confounding effect on muscle water content [56]. Again, MuscleSound^®^ software was used to interpret the ultrasound images and was compared to that of the suction-modified percutaneous needle biopsy procedure. The results from this study were unable to validate the use of ultrasound technology to estimate muscle glycogen or increases/decreases in these stores across a range of scenarios including exercise-depletion, normalized stores, carbohydrate loading, and concomitant creatine loading [56]. Although the use of non-invasively measuring muscle glycogen content with a portable deceive is appealing, more research and development is needed to ensure valid and reliable results in the world of elite sport under varying environmental conditions. However, this is hopeful for the future and if corrections are made, this technology would have extensive application in applied sports nutrition.

To critically compare the above methods for glycogen detection, one may refer to Table 1 below, which provides a comparison of the techniques in terms of portability, accuracy, time needed to perform measurements, size, and cost. 

### 4.5. Advances in Sensor Technology to Detect Physiological Markers

**Figure 9:**
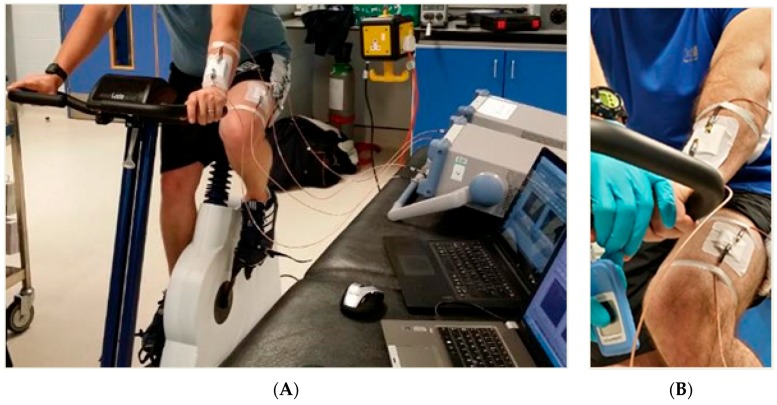
(**A**) Experimental setup, showing participant on ergometer with sensors attached; and appropriate data acquisition hardware: (**B**) illustrates placement of sensors both arm and leg during a blood lactate exercise protocol.

Although currently there are not any sensors with the specialised capability of detecting muscle glycogen non-invasively, it is key to highlight similar efforts at the forefront of current technology that have enabled previously invasive methods of personal health monitoring and optimal performance tracking to become real-time and non-invasive. Furthermore, technology is constantly improving, it is estimated in the next few years such equipment will be available on the market to measure such parameters. Electromagnetic (EM) waves are waves of energy that travel through a vacuum at the speed of light, which is approximately *c* = 3 × 10^8^ m/s [58,59]. Microwave detection in physiology is an emerging new field offering a vast range of applications. Microwave sensors provide a real-time non-invasive method of analysis, which is cost-effective, robust and has many practical applications from a sport and athletic stand point, yet there has been no successful attempt within this industry. This revolutionary system eliminates the need for blood samples, muscle biopsies, and other invasive methods for the selected parameters by being placed over the blood vessels on the skin (Figure 9a). Microwave sensor technology has the hope of being able to detect an array of different parameters, these may include (but are not limited to): muscle glycogen, blood lactate (Figure 9b), blood oxygenation, water content, and muscle proteins consecutive to exercise-induced muscle damage. The above parameters are monitored by sending electromagnetic waves through the skin and sensing reflection, this technique uses extremely low levels of radiation (three to four orders of magnitude lower than that of mobile-phones) avoiding any exposure to harmful radiation.

Previous research using this technology has demonstrated the ability to detect a variety of different substances such as glucose at physiological levels as well as lactate in water and types of oils [60,61,62]. The study by Goh et al. [63] used this microwave sensing platform to successfully detect lactate in cerebrospinal fluid. This shows application of this technology could provide masses of benefits to a variety of different practitioners within the world of sports and performance who want to be able to quantify previously difficult performance markers both in the labs and in the field. The sensor uses electromagnetic waves of 300 MHz to 300 GHz, the sensor is based on conductivity (ability of a material to conduct an electric current) and permittivity (which depends on the dielectric constant of the material) [64]. The use of a non-invasive sensor allows for durability, simple design, portability, and ability for real time analysis [65,66,67]. Although this technique is a promising concept, further research and development is needed before the sensors will influence the world of sports and exercise science. 

In recent years, there have been successful attempts in the development of sensors that process the ability to detect glucose levels non-invasively for patients involved in diabetes management. The prerequisite for non-invasive detection was due to the need in avoiding the complex, costly, and painful nature of conventional (invasive) glucose monitoring. Glucose monitoring is of special importance because of its involvement in the human metabolic process, giving promise to the future of non-invasive glycogen sensors. Jiang et al. [68] developed a sensitive glucose biosensor designed by the immobilization of Os-complex mediator and glucose oxidase on the electrode surface. The biosensor successfully determined the glucose extracted from the skin by reverse iontophoresis (the transport of glucose outward from the skin) and demonstrated a relative amperometric reaction to accumulative subcutaneous glucose levels [69]. This is one of many examples of successful non-invasive glucose monitoring which have come about only in the last decade which alongside reverse iontophoresis [70] include, bioimpedeance spectroscopy [71], ultrasound/electromagnetic and heat capacity [72], laser microporation [71], and the Prelude^®^ SkinPrep [73] system. There are many other systems which are still lacking well-documented clinical trials but give assurance in the future of an affordable non-invasive glycose monitoring [74]. Although non-invasive detection of glucose is a credit to advancing personal health care monitoring technology, there is still a lot of research needed to develop similar devices for glycogen. Glucose is a much simpler molecule than glycogen, unlike glucose, glycogen is insoluble in water and cannot pass in and out of cells until glycogenolysis occurs which breaks it down into much smaller soluble units. Despite this, the use of the above techniques show promise, with the necessary essential development could provide a method suitable for glycogen detection. 

## 5. Conclusions and Future Research Directions

Muscle biopsies in combination with histochemical assay remains the preferred method of choice to measure muscle glycogen despite its invasive method. This will remain so until an alternative device is available which reaches a high standard of accuracy, allowing for portable, real-time, and non-invasive assessments. MRS is leading the way with the non-invasive measurements of glycogen, when MRS and muscle biopsy samples are used in conjunction, the biopsy can be used to measure other metabolic variables such as enzymatic activities leaving MRS to detect glycogen levels. This combination is rewarding for scientists and professionals who can then develop a strategy to optimize athletic performance. To date, research into the measurement of muscle glycogen using MRS focuses mainly on the clinical side rather than athletic performance. However, MRS isn’t practical when monitoring athletic performance parameters due to the costs involved and the issue that MRS is conducted on a MRI machine usually within a hospital setting. Although data from musculoskeletal high frequency ultrasound initially showed promising results, further research has shown the flaws, further development is required before this technique can be applied to the ever-changing circumstances of a professional athlete’s regime. Electromagnetic sensors being able to measure muscle glycogen non-invasively is a serious possibility. Future research needs to ensure accurate results in conjunction to the gold standard technique. Providing a number of different muscle glycogen manipulation strategies within the methodology will ensure measurements are accurate under a variety of conditions avoiding any limitations. 

Invasive measurement techniques, such as those requiring a lancet to perform fingertip prick tests (e.g. for diabetes or lactate monitoring) involve have a place in the market until such time that non-invasive replacements are both widely available and inexpensive; they will enable greater frequency of measurement and reduce the associated pain in the measurement procedure. Similarly, the need for non-invasive methods is present in the world of elite human performance to provide accurate nutritional guidelines for optimising access to glycogen stores in real time, thus avoiding the need for biopsies. 

With recent studies researching the effects of different levels of CHO levels from ‘real life’ perspectives within the world of sport, the need for real time non-invasive measuring equipment is ever more valuable. This would solve many of the issues faced by scientists and coaches using invasive equipment out of the laboratory. The research that would follow would allow measurement of the athlete’s ability to repeat glycogen super compensation protocols, this ability is of profound interest to elite competitors such as professional cyclists and team sport athletes who commonly perform multiple sessions of training and competition each week.

## Figures and Tables

**Table 1:** Assessment of current techniques to detect muscle glycogen in athletes.

Glycogen Assessment Techniques	Portable?	Accuracy	Real-Time?	Non-Invasive?	Size?	Time of Measurement	Cost
Modified Bergström Muscle Biopsy/Assay	Yes	High	No	No	The Bergström needle (5 mm)	days	Low
Magnetic resonance spectroscopy	No	High	No	Yes	Height 200 cm approx.	min/h	High
					Width 199 cm approx.		
Musculoskeletal high frequency ultrasound	Yes	Low	Yes	Yes	Height 121 cm approx.	min	Moderate
					Width 40 cm approx.		
